# Developmental data for several human mitochondrial DNA (mtDNA) long amplification targets

**DOI:** 10.1016/j.dib.2020.106007

**Published:** 2020-07-09

**Authors:** Mark F. Kavlick

**Affiliations:** Research and Support Unit, Laboratory Division, Federal Bureau of Investigation, 2501 Investigation Parkway, Quantico, VA 22135, USA

**Keywords:** Mitochondrial DNA, MtDNA, Quantitative PCR, QPCR, Hydrolysis probe, DNA fragmentation

## Abstract

Candidate long mtDNA targets ∼300 bp in length were identified on the revised Cambridge mtDNA reference sequence using Primer Express software (Applied Biosystems) with modified default analysis settings. The primer and hydrolysis probe sequences for the resultant three (3) targets were queried in the Mitomap database [1] to avoid common single nucleotide polymorphisms (SNPs) which, if present in a sample, could reduce binding to template and therefore result in inefficient amplification. Primers and probes identified by Primer Express, some synthesized degenerate to mitigate the presence of certain SNPs, were utilized in a Fast Advanced Master Mix (Applied Biosystems) reaction which was amplified on a 7500 Real Time PCR System using HID Real Time PCR Software v1.2 (Applied Biosystems) to collect and analyze the qPCR data. QPCR reaction conditions and software analysis settings were optimized and modified to yield efficient amplification and robust results. QPCR experiments were exported into Excel (Microsoft Corp.) for additional analyses and evaluation. The data was used to develop a triplex qPCR method, which includes amplification of one of the long targets, to quantify and assess degradation of human mtDNA, the results of which were previously published [2]. That triplex method also incorporated an internal positive control to test for the presence of amplification inhibitors in the sample [3]. The data presented herein may be used to develop alternative amplification methods for user-specific biomedical applications.

Specifications table

**Table:** 

Subject	Biochemistry, Genetics and Molecular Biology (General)
Specific subject area	Quantitative and Qualitative Assessment of Mitochondrial DNA via Quantitative PCR (qPCR)
Type of data	QPCR sequence detection system (.SDS) files Spreadsheet (CSV and XLSX) files Tables Graphs Figures
How data were acquired	Primer Express Software, v2.0 and v3.0, Applied Biosystems, Foster City, CA Quantitative PCR utilizing: 7500 Real Time PCR System, Applied Biosystems HID Real Time PCR Software, v1.2, Applied Biosystems
Data format	Raw Analyzed
Parameters for data collection	Parameters for data collection using the HID Real Time PCR Software v1.2 (Applied Biosystems) followed the manufacturer's recommendations with the *automatic baseline* setting and cycle threshold setting at 0.2 ΔRn.
Description of data collection	Data were collected for qPCR reactions containing the Fast Advanced Master Mix using HID Real Time PCR Software v1.2 on a 7500 Real Time PCR System (Applied Biosystems) following the manufacturer's recommendations and imported into Excel software (Microsoft Corp.) for processing and analyses.
Data source location	Institution: Federal Bureau of Investigation, Laboratory Division, Research and Support Unit City/Town/Region: Quantico, Virginia Country: United States
Data accessibility	The data associated with this article is hosted in a public repository. Repository name: Mendeley Data Data identification number: doi:10.17632/66bgm2cbyc.1 Direct URL to data: https://data.mendeley.com/datasets/66bgm2cbyc/draft?a = 836a5476–0603–429e- 9a00-ab6f6676ba70
Related research article	Mark F. Kavlick Development of a triplex mtDNA qPCR assay to assess quantification, degradation, inhibition, and amplification target copy numbers Mitochondrion [2] DOI: 10.1016/j.mito.2018.09.007

## Value of the data

• This article provides useful developmental data for three long mtDNA targets for highly efficient hydrolysis probe qPCR, as originally developed to assess fragmentation of mtDNA [2]. The data includes important information on target mtDNA sequence variation, amplification primer and hydrolysis probe optimizations, and specificity for human mtDNA.
• The biomedical, ancient DNA, and forensic science research communities may benefit from this data by acquiring developmental information relevant to these long mtDNA targets for use in alternative user-specific assays, methods, and applications, whether for general or target-specific mtDNA studies.
• The information may be used for development of alternative methods such as quantification by intercalating dye qPCR, quantification of intact mtDNA, assessment of mtDNA degradation, PCR amplification, and sequencing of specific mtDNA regions.
• Additional value of these data includes 1) identification of similar-length targets both within and outside the mtDNA 5 Kb “common deletion” region [4] for potential use in studies of aging and disease and 2) identification of a probable mtDNA target (long set #2) for quantification of chimpanzee mtDNA.

## 1 Data

### 1.1 Identification of candidate long mtDNA targets

**Fig. 1:**
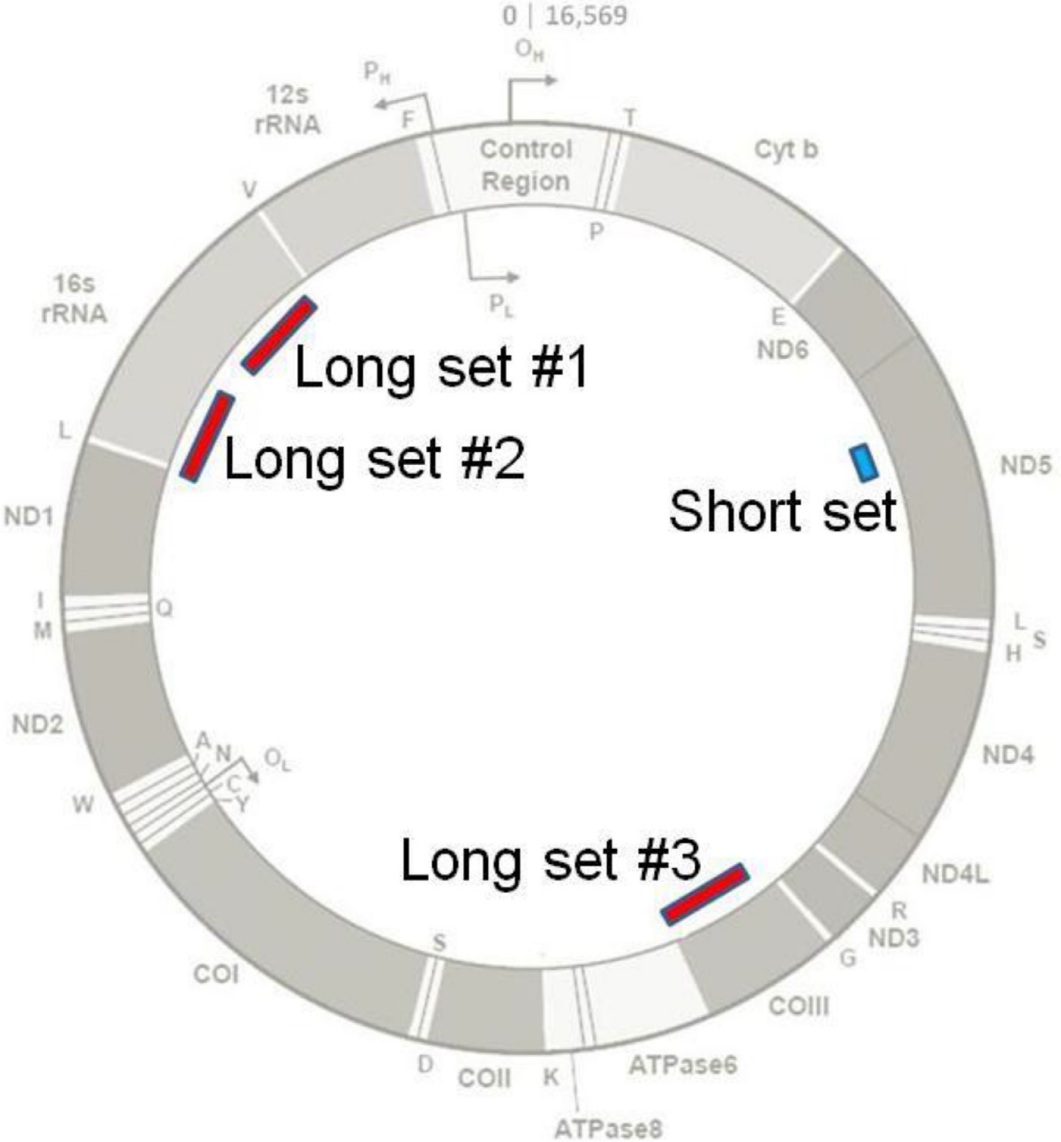
Locations of candidate long mtDNA target primer and probe sets. This diagram of the circular mtDNA genome illustrates the relative positions and lengths of the three candidate long targets to that of a short target set. Shown are the mtDNA base positions (top, center), the Control Region which contains three hypervariable regions, gene designations (inner and outer rings), origins of replication for the heavy (OH) and light (OL) strands, and promoter locations for the heavy (P_H_) and light (P_L_) strands. The blue bar depicts a short 105 bp mtDNA target [5,6] and the red bars represent the three candidate long mtDNA targets described here, each ∼300 bp in length, for potential use in assessing mtDNA degradation in a triplex qPCR assay. Counter-clockwise from the top, the long targets were 1) a 316 bp amplicon wholly within the 16s rRNA gene, 2) a 273 bp amplicon mostly within the 16s rRNA gene and extending into the “L” (tRNAleu) gene, and 3) a 265 bp amplicon wholly within the cytochrome c oxidase subunit III gene. The figure is a modification of that found at www.mitomap.org/MITOMAP[1] and is reproduced with permission.

Three (3) separate long mtDNA target sets were identified which ranged in length from 265 to 316 bps, each with its own forward primer, reverse primer, and probe, and are referred to herein as long set #1, long set #2, and long set #3 (Fig. 1). These candidate sets were queried for the presence of any common single nucleotide polymorphisms (SNPs) in the Mitomap database [1], defined here as those having a frequency ≥0.5%, among the various primer and probe sequences. This analysis identified a total of six SNPs which ranged in frequency from 0.5% to 14.4% from among the database's set of 50,175 full length mtDNA sequences collected from GenBank [7] on January 1, 2020. To ensure that the vast majority of human mtDNA sequences encountered in practice would be amplified by these sets, the relevant primers and probes were synthesized with the appropriate degenerate bases (Table 1).

**Table 1:** Three candidate long mtDNA amplicon primer and probe sets.

Target sequences were also subjected to searches at the National Center for Biotechnology Information (NCBI) using the Basic Local Alignment Search Tool for nucleotides (BLASTN) against the RefSeq Genome Database (refseq_genomes) [8] to detect potential homology to non-human genome sequences. Those results revealed no complete homology for all human long target mtDNA sets with any other species’ DNA, except for long set #2 which exhibited full homology with chimpanzee DNA (data not shown).

### 1.2 QPCR amplification optimizations

Pre-optimized baseline amplification efficiencies for the three long target singleplex primer and probe sets were observed to be >89% for each set (data file 070914.sds). Next, the three (3) long target sets (VIC-labelled probes), as well as a short target set (FAM-labelled probe), and a custom IPC target set (NED-labelled probe) - the latter two being targets which have been incorporated in a triplex qPCR assay previously described [2], five (5) singleplex assays in total - were optimized with respect to primer (data files 071614.sds and 071614_2.sds) and hydrolysis probe (data file 071814_3.sds) concentrations. The optimal primer and probe concentrations for the three long mtDNA targets are identified in Table 1.

**Fig. 2:**
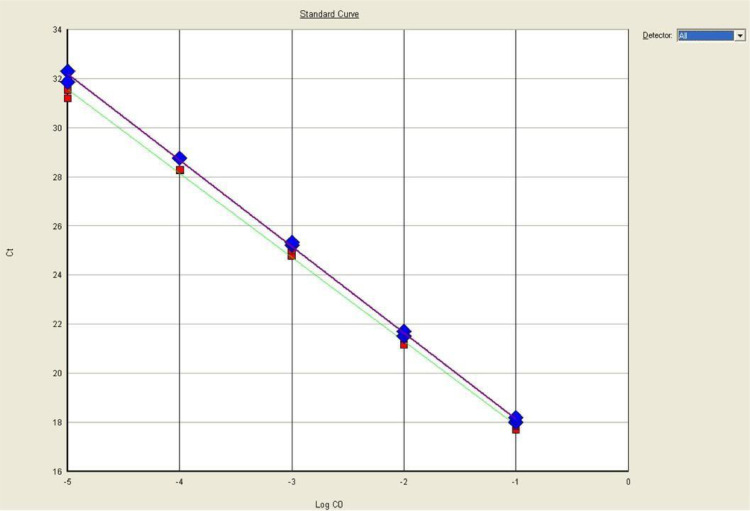
Standard curves for mtDNA short and long set #1 targets. Shown are the standard curves for a representative triplex experiment, of five such experiments, in which HL60 DNA dilutions were used as the standard. Short set (blue diamonds) and long set #1 mtDNA targets (red squares) were assayed in duplicate and exhibited similar slopes, and thus similar efficiencies which averaged 92.8 and 95.7%, respectively. Not shown is the NED-signal since the IPC system does not amplify HL60 DNA.

The optimal primer and probe concentrations, as identified in each of the singleplex assays, were used for the three (3) long and short target duplex assays as well as the three (3) triplex assays, with the custom IPC added (see data files 072514.sds, 073014.sds, 073114.sds, 080614.sds, and 080614_2.sds). With this approach, no substantial change in the amplification efficiencies was evident (duplex efficiencies not shown; triplex efficiencies shown in Table 2). Among the triplex assays, long set #1 appeared to be most reproducibly efficient within a range of 1.5% while the short target set was similarly reproducible (1.7%). In addition, the difference between the mean long set #1 and mean short set efficiencies was only 2.9%, with the former being slightly more efficient than the latter (Fig. 2).

**Table 2:** Long and short mtDNA target amplification efficiencies (triplex qPCR assays, with IPC).

	Exp1	Exp2	Exp3	Exp4	Exp5	MEAN	Difference of Means	Min	Max	Range
Short Set Mean	92.6%	91.3%	92.4%	92.6%	92.7%	92.3%				
Short Set with Long Set 1	93.2%	91.7%	92.7%	93.4%	92.9%	92.8%		91.7%	93.4%	1.7%
Long Set 1	95.9%	95.4%	96.3%	95.9%	94.8%	95.7%	2.9%	94.8%	96.3%	1.5%
Short Set with Long Set 2	91.4%	92.2%	93.2%	93.2%	92.8%	92.6%		91.4%	93.2%	1.8%
Long Set 2	94.7%	98.8%	97.4%	96.0%	95.7%	96.5%	3.9%	94.7%	98.8%	4.1%
Short Set with Long Set 3	93.1%	89.9%	91.4%	91.1%	92.5%	91.6%		89.9%	93.1%	3.2%
Long Set 3	95.6%	93.5%	94.3%	92.4%	94.3%	94.0%	2.4%	92.4%	95.6%	3.2%

### 1.3 Species specificity and RHO zero cell line DNA testing

All three candidate triplex assays were further evaluated for cross-reactivity to non-human species’ DNA, and for activity to human A549 cell line DNA, and human Rho Zero 143B cell line DNA, the latter purportedly depleted of mtDNA but not human *nuclear* DNA sequences of mitochondrial origin (NUMTs) [9,10]. DNA from up to 32 non-human species was tested, including that of chimpanzee and other primates, mammals, fish, birds, fungi, and bacteria (see data files 091614.sds, 091614.csv, 091614_1.sds, 091614_1.csv, 091714.sds, 091714.csv, 110514.sds, 110514_2.sds, and 110514_3.sds). The results showed that (see data file Species_Specificity_Tables_0720.xlsx) for the long set #1 and long set #3 triplex reactions, little cross-reactivity to other species’ DNA was observed, chimpanzee DNA included. However, the long set #2 triplex reaction exhibited high cross-reactivity to chimpanzee DNA, a result which was not unexpected since the human long set #2 target sequence was found to be fully homologous with the chimpanzee mtDNA sequence in the NCBI database. Because of this observed cross-reactivity, further species specificity testing for the long set #2 triplex was limited in scope; however, virtually no additional cross-reactivity was observed for the set #2 triplex. Amplification of Rho zero DNA by the three long mtDNA amplification sets was negligible. It is also noteworthy that the short 105 bp mtDNA target [5,6] did not appreciably amplify chimpanzee DNA within any of the three triplex reactions.

### 1.4 Selection of triplex assay for further development

Taken together, all three triplex reactions for human mtDNA quantification and determination of fragmentation were deemed suitable for further development and testing. However, the long set #1 triplex reaction alone was chosen for further triplex assay development due to certain favorable aspects of that assay compared to the other two (Table 3). This included the location of long set #1 within the mtDNA genome, i.e., outside of the major mtDNA common deletion [4] and therefore an alternative location to the short 105 bp target, a minimal number of reported SNPs, a greater consistency of amplification efficiency, lack of cross-reactivity to chimpanzee DNA, and a higher ΔRn value, i.e., greater fluorescent signal, relative to the other two long sets. The fully developed triplex assay has been reported elsewhere [2].

**Table 3:** Attributes of three candidate long set targets and their respective triplex assays.

## 2 Experimental design, materials, and methods

### 2.1 DNA samples

Non-human vertebrate DNA samples were purchased from Zyagen Laboratories (San Diego, CA) with the exception of chimpanzee DNA which was obtained from the NIGMS Human Genetic Cell Repository at the Coriell Institute for Medical Research (Camden, NJ): # NS06006. Human HL60 cell line DNA, human A549 cell line DNA, bacterial DNA, and fungal DNA were purchased from the American Type Culture Collection (ATCC; Manassas, VA). Human rho zero (ρ0) 143B cell line DNA, which is mtDNA depleted [9,10], was a component of the NovaQUANT Human Mitochondrial to Nuclear DNA Ratio Kit (EMD Millipore Corp., San Diego, CA).

### 2.2 Identification of candidate targets

To identify long candidate mtDNA sequence targets, the complete 16,569 nucleotide FASTA sequence of the revised Cambridge Reference Sequence (rCRS) [11], GenBank sequence NC_012920, was selected as the template to identify suitable amplification primers and fluorogenic hydrolysis probes for assessing DNA degradation in a triplex assay. This sequence was queried with Primer Express software, v2.0 and v3.0, (Applied Biosystems, Foster City, CA) using the default analysis parameters with the exception of target amplicon length which was increased to the range of 250–350 bp, or ∼3x the length of the short 105 bp mtDNA target described previously [5,6].

### 2.3 QPCR

The sequences and concentrations for the qPCR primers and probes are described in Table 1 and elsewhere [2]. Primers were HPLC-purified (Integrated DNA Technologies, Coralville IA) and reconstituted with 10 mM tris 0.1 mM EDTA buffer, pH 8 (TE) as 100 µM stocks. Probes were FAM-, VIC-, or NED-labelled, each containing a 3′ minor groove binder non-fluorescent quencher (MGB-NFQ) and supplied as 100 µM stocks (Applied Biosystems).

The short target qPCR standard sequence corresponded to nucleotide positions 13,288 ∼ 13,392 in the ND5 gene of the mtDNA rCRS, i.e., the target sequence, plus five additional base pairs at both the 5′ and 3′ ends and a signature sequence, both for quality control [5,6]. The standard consisted of two complementary, PAGE-purified synthetic oligonucleotides (Ultramers; Integrated DNA Technologies) which were diluted in TE to achieve serial 10-fold dilutions from 10^7^ to 10^1^ copies/µL. Briefly, paired forward and reverse oligonucleotides were separately reconstituted in TE, quantified by absorbance at 260 nm using the extinction coefficients 1,082,000 and 1,138,100 L/(mole•cm), respectively, then adjusted to 2 µM. The adjusted oligonucleotides were mixed in equal proportions to generate a 1 µM ds, primary standard stock. On applying Avogadro's constant, the primary stock was further diluted with TE to generate the first standard, 10^7^/µL, which was serially diluted 10-fold to generate the remaining standards in the dilution series: 10^6^, 10^5^, 10^4^, 10^3^, 10^2^, and 10^1^ copies per µL.

The novel IPC template similarly consisted of two complementary, PAGE-purified synthetic oligonucleotides (Ultramers; Integrated DNA Technologies) which were reconstituted in TE, quantified by absorbance at 260 nm using the extinction coefficients 628,700 and 621,800 L/(mole•cm), respectively, prepared as a duplex in equimolar proportions, and diluted to 1,250 copies/µL [2,3]. HL60 DNA (ATCC), diluted to 20 pg/µL in TE, served as the calibrator in triplex reactions [2].

QPCR assays were performed using 2 µL of sample DNA, standard DNA, TE as a no template control (NTC), or HL60 calibrator, in a 20 µL reaction containing 10 µL of TaqMan 2X Fast Advanced Master Mix (Applied Biosystems) following the manufacturer's recommendations [12,13] in a hybrid, relative-absolute quantification assay [2,14].

### 2.4 QPCR amplification optimizations

The three long target primer and probe sets were first separately tested in singleplex qPCR reactions using a dilution series of HL60 DNA as a standard to determine the baseline efficiency of the respective reactions. Next, each of the five (5) singleplex assays - the short target set (FAM-labelled probe), each of the three (3) long target sets (VIC-labelled probes), and the custom IPC set (NED-labelled probe) - were optimized with respect to primer and hydrolysis probe concentrations, using fixed HL60 DNA or IPC template DNA concentrations as appropriate, according to the recommendations of the probe manufacturer, Applied Biosystems [15]. Briefly, for each amplification set, forward and reverse primer concentrations were varied at 50, 300, and 900 nM final concentrations to achieve a minimal Ct value. Probe concentrations were varied at 50, 100, 150, 200, and 250 nM final concentrations to achieve a maximal ΔRn value. Finally, the three long target primer and probe (VIC-labelled) set concentrations, thus optimized, were separately tested in duplex reactions, which included the short target set (FAM-Iabelled probe), and in triplex qPCR reactions, which also included the custom IPC set (NED-labelled probe). These reactions utilized HL60 DNA as the standard so that efficiencies for the long sets could be determined.

### 2.5 Species specificity and rho zero cell line dna testing

DNA from up to 32 non-human species as well as from human HL60, A549, and 143B Rho zero cell lines was tested with the triplex qPCR assays, this included DNA from chimpanzee and other primates, mammals, fish, birds, fungi, and bacteria (described above). Since the assay was designed for human specificity, the non-human test DNA samples were not expected to appreciably amplify. However, cross-reactivity, when present, was manifested by a positive quantity for the short target. In such cases, the non-human DNA “quantity” was divided by the quantity determined for human HL60 DNA, after normalization, to derive the percent cross reactivity of DNA from that species to the short target, i.e, percent of human (see data file Species_Specificity_Tables_0720.xlsx). Percent cross-reactivity to the long targets, also as a percent of human, was determined using the delta delta Ct method [14] with HL60 as the calibrator and relative comparison to the respective short target percent of human cross-reactivity.

## Declaration of Competing Interest

The author declares no known competing financial interests or personal relationships that could have appeared to influence the work reported in this paper.
